# Low-grade fibromyxoid sarcoma incidentally discovered as an asymptomatic mediastinal mass: a case report and review of the literature 

**DOI:** 10.1186/s13256-020-02605-4

**Published:** 2021-02-02

**Authors:** Mir Ibrahim Sajid, Sidra Arshad, Jamshid Abdul-Ghafar, Saulat Hasnain Fatimi, Nasir Ud Din

**Affiliations:** 1 Medical College, Aga Khan University, Karachi, Pakistan; 2 Department of Pathology and Laboratory Medicine, Aga Khan University Hospital, Karachi, Pakistan; 3 Department of Pathology and Clinical Laboratory, French Medical Institute for Mothers and Children (FMIC), Kabul, Afghanistan; 4 Section of Cardiothoracic Surgery, Department of Surgery, Aga Khan University Hospital, Karachi, Pakistan

**Keywords:** Low-grade fibromyxoid sarcoma, Mediastinum, Aggressive behavior

## Abstract

**Background:**

Low-grade fibromyxoid sarcoma (LGFMS) is a rare tumor characterized by bland histological features and aggressive clinical course. The most common anatomic locations of occurrence are the lower extremities, thorax, inguinal area, and upper limbs. Primary mediastinal sarcomas are even rarer. To the best of our knowledge, only seven cases of primary mediastinal LGFMS have been reported in the literature. Here, we report a case of primary mediastinal LGFMS.

**Case presentation:**

A 26-year-old Pakistani man presented with fever and vomiting for the past 2 months. On a routine chest x-ray, a mediastinal mass was incidentally found. Computed tomography (CT) scan showed a large circumscribed lobulated soft tissue density mass lesion in an anterior mediastinum. Grossly, the resected mass measured 17.0 × 12.0 × 11.0 cm. The cut surface was gray white with a whorled-like appearance and foci of calcification and cystic changes. Histologically, a spindle cell lesion was seen with alternating myxoid and hyalinized areas. The shaped cells were arranged in bundles. Immunohistochemical staining showed positive reactivity patterns with MUC4 and focally for epithelial membrane antigen (EMA). The diagnosis was confirmed as LGFMS. The patient is free of symptoms and recurrence 22 months after the surgery.

**Conclusion:**

In conclusion, we report a rare case of primary mediastinal LGFMS in a young male patient that was discovered incidentally. Our patient is on regular follow-up to look for evidence of recurrence as these tumors are prone to recurrences.

## Introduction

Low-grade fibromyxoid sarcoma (LGFMS) is a rare tumor with generic histologic characteristics and intense clinical progression. This tumor is found predominately in the extremities, both upper and lower, inguinal region, and thorax [1]. On histological examination, this tumor is found to consist of alternating fibrous and myxoid areas, arranged in a spiral fashion with fibroblast spindle cells that appear benign. Primary mediastinal sarcomas are rarer than ever. To the best of the authors’ knowledge and the literature search performed, only seven cases of primary mediastinal LGFMS have been reported [2–7], and this is an additional rare case of primary mediastinal LGFMS with typical histological findings, which was confirmed by immunohistochemical (IHC) stains [8].

Since this was a retrospective observational study and did not involve actual patients or patient’s images, ethical approval was not sought for this study.

## Case presentation

**Fig. 1:**
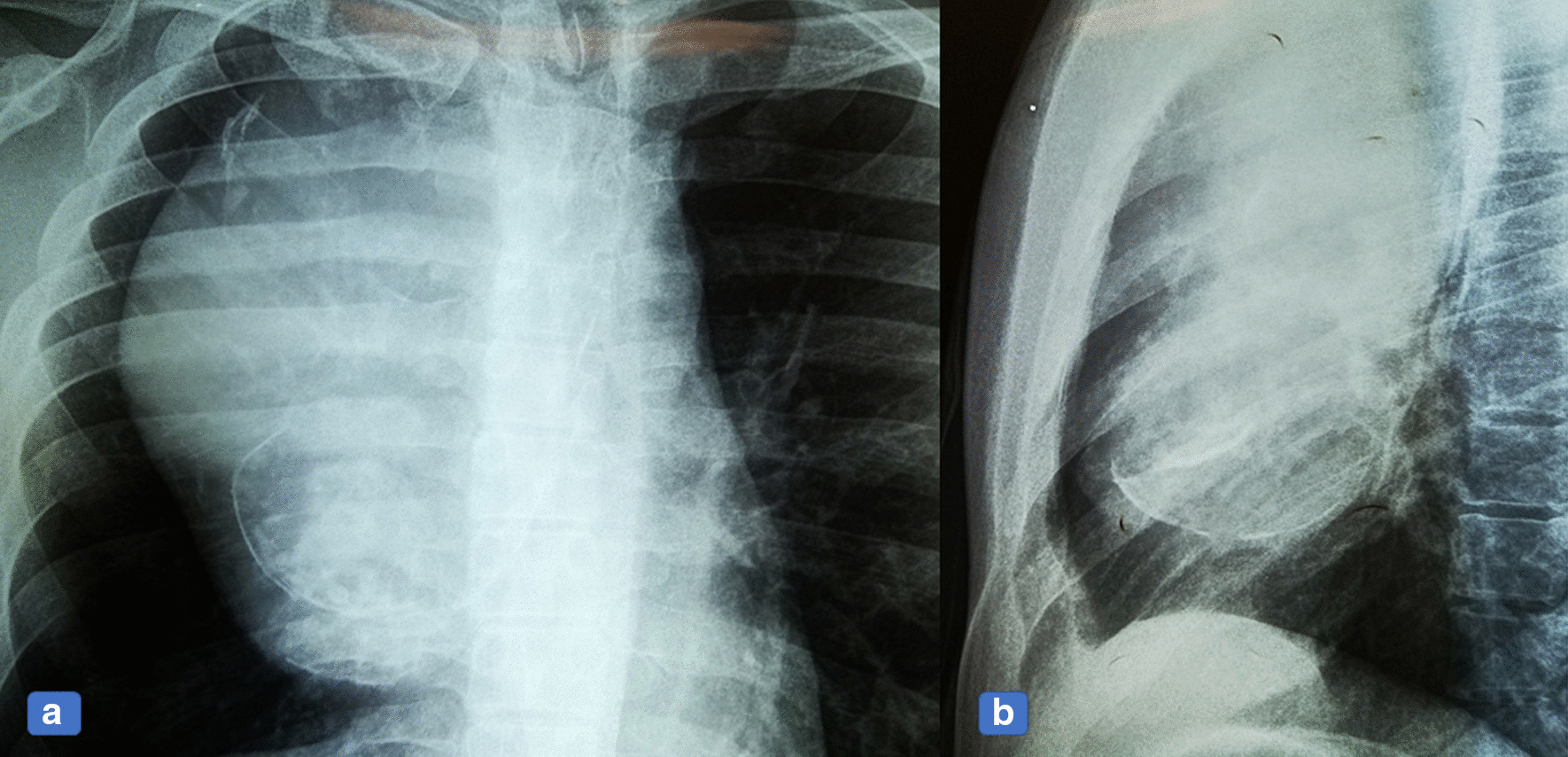
**a** Chest x-ray, Anterior Posterior (AP) view, showing a large, lobulated, soft tissue density mass with internal calcification. It forms an obtuse angle causing superior mediastinal widening, more on the right side. **b** Lateral chest x-ray shows obliteration of the retrosternal space.

**Fig. 2:**
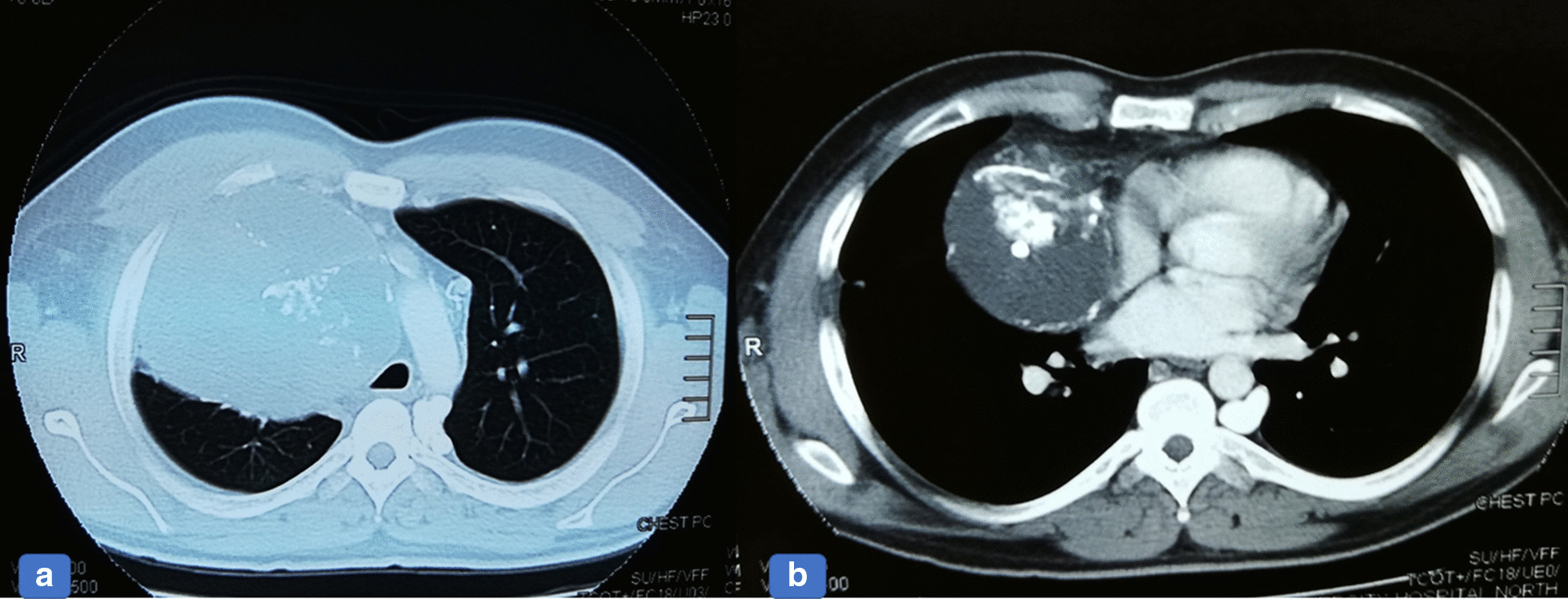
**a**, **b** Axial images of the chest-lung computed tomography and mediastinal window show well-defined rounded low attenuation mass with internal coarse calcification.

A 26-year-old Pakistani man presented with fever and vomiting (non-bilious and non-bloody). He had been under treatment in another hospital for the past 2 months. Due to progressive worsening of symptoms, he was kept as an inpatient with a working diagnosis of acute hepatitis A and jaundice and was managed accordingly. A mediastinal mass was incidentally found during a routine chest X-ray (Fig. 1a, b). A computed tomography (CT) scan was then ordered to characterize the lesion. The CT scan showed a large circumscribed soft tissue density lesion in the anterior mediastinum, extending laterally in the right hemithorax abutting the anterior and right lateral chest wall without evidence of infiltration (Fig. 2a, b). Superiorly, it was extending into the root of the neck, abutting the posterior cortex of the medial end and compressing the superior vena cava, displacing the ascending and arch of the aorta as well as its major branches, trachea, and esophagus toward the left side. Multiple foci of coarse calcifications were also noted within the lesion. The lesion measured 16.9 × 12.8 × 11.0 cm. The described imaging features suggested the possibilities of a germ cell tumor or hydatid cyst. For surgical resection of the mass, the patient was referred to our hospital. The patient was briefed about the procedure and was taken to the operating theatre for right thoracotomy and tumor resection with an American Society of Anesthesiologists (ASA) score of II. Intraoperatively, a 20.3 × 12.7 cm solid mass filled with green cystic fluid was visualized extending to the right hemothorax. It was not adherent to the lung but had definite adhesions with the hilar structures.

**Fig. 3:**
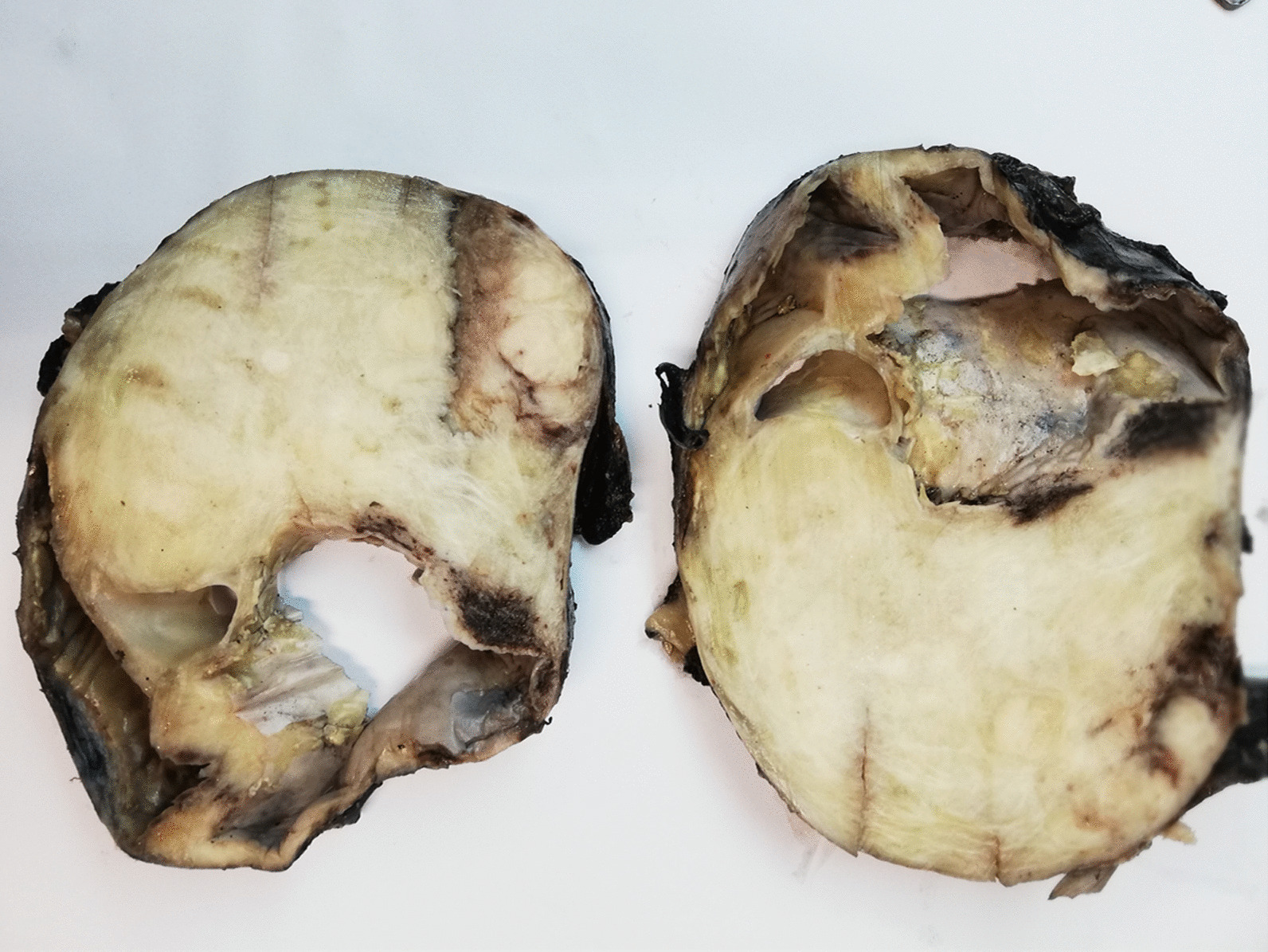
Gross examination. Cut surface shows a gray-white, firm, solid tumor with cystic areas

**Fig. 4:**
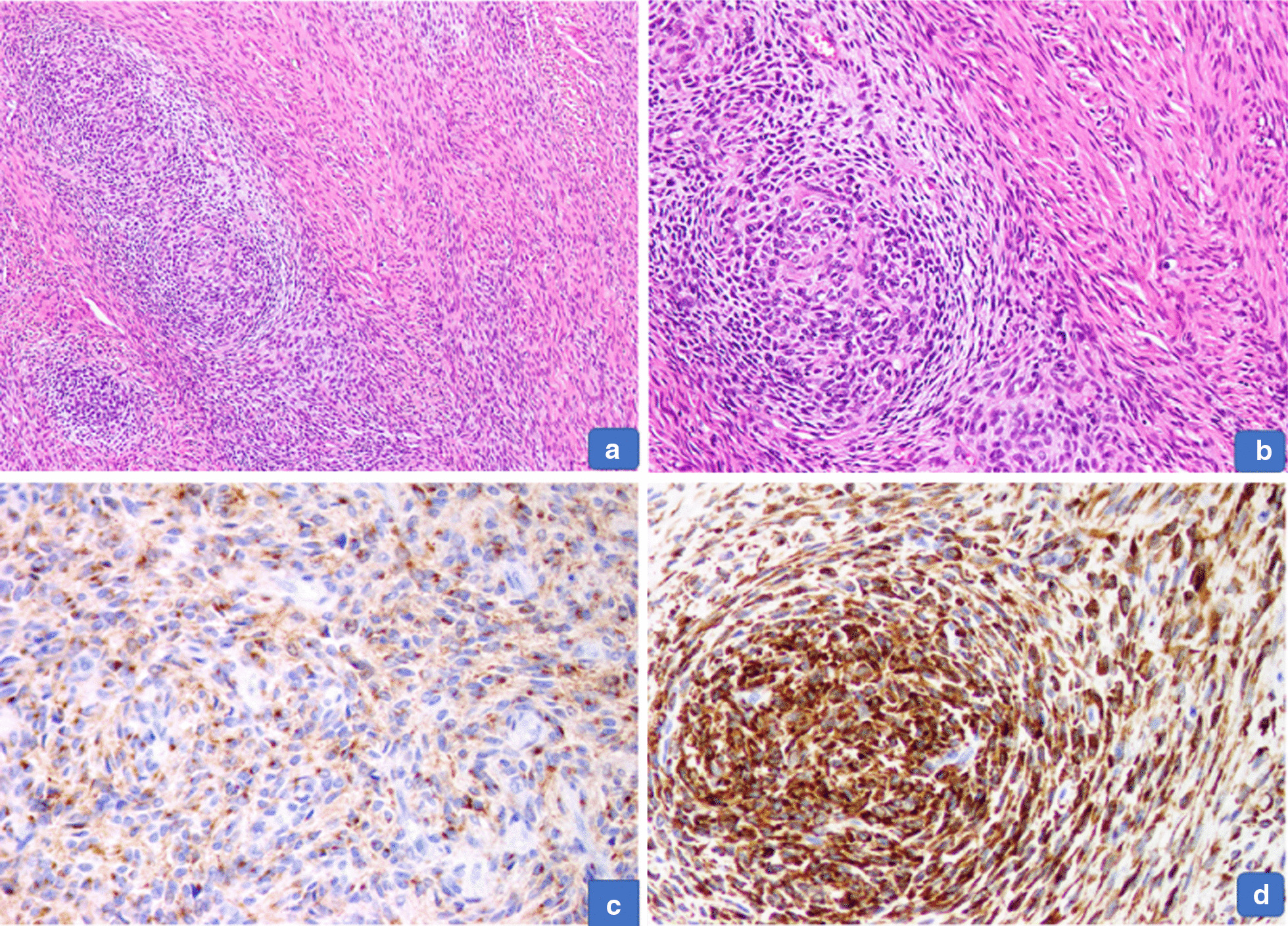
**a**, **b** Low and intermediate power shows a tumor with alternating cellular myxoid and fewer cellular collagenized areas. **c** EMA positivity in tumor cells. **d** Diffuse strong MUC 4 positivity in tumor cell

Grossly, the resected mass was encapsulated and measured 17.0 × 12.0 × 11.0 cm. The cut surface was gray white with a whorled-like appearance and foci of calcification and cystic areas (Fig. 3). Histologically, a spindle cell lesion is seen with alternating myxoid and hyalinized areas. The shaped cells were arranged in bundles. In areas, myxoid hypocellular areas were identified with few intervening dilated blood vessels and dense collagen bundles (Fig. 4a, b). Multiple foci of calcifications were visualized at the periphery with collagen rosettes. A few areas also showed a sprinkling of inflammatory cells, predominantly plasma cells. IHC staining was performed, which showed positive reactivity patterns with MUC4 and focally for epithelial membrane antigen (EMA) (Fig. 4c, d). CD34, anti-smooth muscle actin (ASMA), desmin, and S100 protein stains were negative. The diagnosis was confirmed as LGFMS.

Postoperatively, the patient was kept nil per oral for 6 hours and started on intravenous analgesics and antibiotics. His chest x-ray showed no residual mass, consolidation, pneumothorax, or pleural effusion. He was sent home in a stable condition on the 4th day of surgery and followed in the clinic for 2 months. The patient is free of symptoms and recurrence 22 months after surgery.

## Discussion

In 1987, Evans first recognized LGFMS as a unique entity seen in young and middle-aged adults, which was initially identified as a slow-growing, asymptomatic tumor of the soft tissue with a deceivingly mild histology but with a higher risk of recurrence and metastasis [9].

The tumor mainly involves the deep soft tissue of the lower extremities, especially the thigh, limb girdle, and trunk. Mediastinal occurrence is very rare (Table 1). In 1999, Takanami *et al.* reported the first case of mediastinal LGFMS in a 35-year-old male, which was primarily misdiagnosed as neurofibroma [2]. After 9 years, it recurred and was then correctly diagnosed as LGFMS. The age range of all patients with reported mediastinal LGMS ranged from 19 to 50 years (mean 35.1 years; median 35 years). Four patients were male, and three patients were female. The tumor size varied between 8 and 23.5 cm (mean 13.3; median 12 cm). Histologically, myxoid areas alternating with collagenized areas composed of spindle cells were observed in four cases, and giant collagen rosettes were also present in three cases. The follow-up period was of variable duration for these cases. Recurrence after 7 and 9 years was observed in two cases. Long-term follow-up is therefore recommended because of the risk of late recurrence and metastasis.

**Table 1:** Clinicopathological features of reported cases of mediastinal low-grade fibromyxoid sarcoma

Reference/year of publication	Age/Sex	Tumor size (cm)	Primary location	Histological findings	Intervention performed	Outcomes
Takanami *et al.* [2] 1999	35/M	9 × 5.5 × 3	Anterior mediastinum	Bland-appearing fibroblastic spindle cells in fibrous and myxoid areas in a whorled pattern	Surgical resection	Recurred after 9 years. Alive without remission or residual symptoms in 2 months of follow-up
Galetta *et al.* [3] 2004	41/M	8 × 4 × 3	Behind the thymus	Vascularized myxoid sarcoma with hyalinized collagenous and spindled-shaped areas	Surgical resection	Alive without remission or residual symptoms in 35 months of follow-up
Jakowski *et al.* [4] 2008	44/F	12	Arose from the right epicardium at the atrioventricular groove adherent to the right ventricular free wall	Admixture of hypocellular myxoid and hyper-cellular spindle cell areas in a collagenous stroma with numerous large hyalinized collagen rosettes	Surgical resection	Alive without remission or residual symptoms in 7 months of follow-up
Gulhan *et al.* [5] 2012	25/F	17 × 13 × 11	Posterior mediastinum, compressing on the anterior mediastinal formations	Lesion formed by spindle-shaped cells was visible in the myxoid and hyalinized stroma. Giant collagen rosettes were also visualized	Surgical resection	Discharged in a stable condition after 11 days and no subsequent follow-up
Maedah *et al.* [6] 2009	**2 cases** 19/F 50/M	23.5 × 10 × 21.5 13 × 13 × 13 **CT measurements** **20 × 10 × 21** **11 × 7.2 × 11.5**	Case 1: Anterior mediastinum Case 2: Right superior mediastinum	Three distinct zones were noted: hyper-cellular zones with islands of collagen fibers, hypo-cellular zones with abundant myxoid stroma, and prominently hyalinized zones	Surgical resection of all the cases	C1: Recurrence of anterior mediastinal mass after 5 years C2 Alive without remission or recurrence in 5 years of follow-up
Pervez *et al.* [7] 2019	32/M	11	Tumor arising from the parietal pleura with attachments to both lung and diaphragm	Tumor was composed of uniform and bland-appearing spindle cells interspersed among large areas of hyalinized rosettes	Surgical resection	No follow-up reported

CT: computed tomography

The histological differential diagnoses of LGFMS include solitary fibrous tumor, low-grade peripheral sheath tumor, neurofibroma, and desmoid fibromatosis. Solitary fibrous tumor with dilated hemangiopericytoma-like vessels and variable stromal collagen exhibits a patternless growth pattern. Tumor cells have homogeneous to ovoid nuclei and an indistinct cytoplasm. In most cases, CD34 is positive, and positivity to STAT6 is diagnostic [10]. Aggressive low-grade peripheral sheath tumor cells show a curly tapered nucleus and cytological atypia and are positive for S100 and SOX10 and negative for MUC4; they show loss of expression for H3K27me. Neurofibroma shows haphazard proliferation of elongated spindle cells that exhibit cytoplasmic eosinophilic processes present in a collagenized or myxoid stroma for stromal mast cells. Tumor cells are positive for S100, SOX10, and CD34 and negative for MUC4. Desmoid fibromatosis shows long, sweeping fascicles with pinpoint nuclei of fibro-/myofibroblastic spindle cells. This tumor is positive for ASMA and beta-catenin and negative for MUC4. Typical histological findings of LGFMS and a positive reaction for MUC4 help to establish a correct diagnosis. Clinical behavior of mediastinal LGFMS seems to be comparable to the LGFMS of extremities.

Approximately 20–25% of all sarcomas show some forms of chromosomal translocations. The majority of LGFMSs have a recurrent balanced translocation between chromosomes 7 and 16, involving the long arm (q) of chromosomes 32–34 and the short arms (p) 11, which results in the union of ‘Fused in Sarcoma’ RNA binding protein’ (FUS) and the CREB3L2 gene [Complex 1]. Translocation of ‘FUS-CREB3L1’ fusion gene is reported in about 5% of the LGFMS cases. The genes CREB3L2 and CREB3L1 play a critical role in codifying transcription factors and contributing to the pathogenesis of the disease. In a study of 14 patients diagnosed with LGFMS, Cesne *et al.* found no association between Complex 1 and the metastatic or recurrent potential of the tumor [11–13].

It is possible to use combined radiotherapy and chemotherapy for locally advanced unrespectable tumors. As with other soft tissue sarcomas, pre- or postoperative radiotherapy with often infiltrative growth patterns can be considered to improve local cancer spread, particularly in the extremities. Some centers use ‘isolated limb perfusion’ (ILP) to salvage limbs during surgery and avoid radiotherapy in a select few patients. Among other localized treatments are cryoablation and radiofrequency ablation (RFA) and cryoablation to treat cancers with more than metastatic lesions [14, 15].

## Conclusion

In conclusion, we report a rare case of primary mediastinal LGFMS that was incidentally discovered in a young male patient. Our patient is regularly looking for evidence of recurrence as these tumors are prone to recurrence. This aggressive sarcoma with a benign appearance should always be present in the differential diagnosis of all benign-looking neoplasms of the spindle cells.

## Data Availability

Data and materials used in this work are available from the corresponding author on reasonable request.

## Abbreviations

LGFMC: Low-grade fibromyxoid sarcoma
IHC: Immunohistochemical
CT: Computed tomography
EMA: Epithelial membrane antigen
ASA: American Society of Anesthesiologists
ASMA: Anti-smooth muscle actin
ILP: Isolated limb perfusion
RFA: Radiofrequency ablation
